# Does Globalization Cause Greenhouse Gas Emissions in Pakistan? A Promise to Enlighten the Value of Environmental Quality

**DOI:** 10.3390/ijerph19148678

**Published:** 2022-07-16

**Authors:** Arif Ullah, Kashif Raza, Muhammad Nadeem, Usman Mehmood, Ephraim Bonah Agyekum, Mohamed F. Elnaggar, Ebenezer Agbozo, Salah Kamel

**Affiliations:** 1 Department of Economics, Divisions of Management & Administrative Science, University of Education, Lahore 54770, Punjab, Pakistan; arifecon@yahoo.com (A.U.); mnadeem.eco@gmail.com (M.N.); 2 UE Business School, Divisions of Management & Administrative Science, University of Education, Lahore 54770, Punjab, Pakistan; kashifrazafsd@yahoo.com; 3 Remote Sensing, GIS and Climatic Research Lab (National Centre of GIS and Space Applications), Centre for Remote Sensing, University of the Punjab, Lahore 54590, Pakistan; usmanmehmood.umt@gmail.com; 4 Department of Nuclear and Renewable Energy, Ural Federal University Named after the First President of Russia Boris Yeltsin, 19 Mira Street, 620002 Ekaterinburg, Russia; agyekumephraim@yahoo.com or; 5 Department of Electrical Engineering, College of Engineering, Prince Sattam Bin Abdulaziz University, Al-Kharj 16273, Saudi Arabia; 6 Department of Electrical Power and Machines Engineering, Faculty of Engineering, Helwan University, Hewlan 11795, Egypt; 7 Department of Big Data Analytics and Methods of Video Analysis, Ural Federal University Named after the First President of 10 Russia Boris Yeltsin, 19 Mira Street, 620002 Ekaterinburg, Russia; eagbozo@urfu.ru; 8 Department of Electrical Engineering, Faculty of Energy Engineering, Aswan University, Aswan 81528, Egypt; skamel@aswu.edu.eg

**Keywords:** environmental degradation, globalization, greenhouse gas emissions, non-linear ARDL, Pakistan

## Abstract

Global environmental issues such as environmental degradation, climate change, and global warming have posed a threat to the global economy, including Pakistan. The primary source of these problems is greenhouse gas emissions. These emissions are the result of human activity. The objective of the study was to investigate the symmetric and asymmetric relationship between globalization and greenhouse gas emissions in Pakistan. The ARDL modern econometric techniques of the time series model were used. Firstly, the stationarity test favors the use of the ARDL model in this study. The BDS test result confirmed that the ARDL model has a non-linearity issue. As a result, the ARDL approach was used to test both the symmetric and asymmetric effect. The results of the asymmetric ARDL model are more robust and reliable than those of the symmetric ARDL model. According to the results of the symmetric ARDL, economic, social, and political globalization have a positive relationship with greenhouse gas emissions in both the short and long run. Furthermore, the long-run results of the asymmetric ARDL model show that positive and negative shocks of economic and political globalization have positive and negative shock effects on greenhouse gas emissions. In the long run, however, the positive shock of social globalization has a negative relationship with greenhouse gas emissions. According to the results of impulse response functions, economic globalization has a significantly more relationship with greenhouse gas emissions than social and political globalization. A policy should be developed that allows only the positive effects of globalization while prohibiting the negative effects of globalization.

## 1. Introduction

Environmental degradation is caused by rapidly expanding anthropogenic operations on a global scale, such as global trade, business, manufacturing, and investments. As a result, before addressing the issue of environmental degradation, a country’s development is unavoidable. Climate disruption, global warming, deforestation, and air pollution are among the major environmental issues. These significant issues arise as a result of Greenhouse Gas (GHG) emissions. The primary GHG emissions elements are carbon dioxide, ozone, methane, nitrous oxide, and water vapor [1,2,3,4,5,6]. Global warming is caused by an increase in the concentration of certain elements in the atmosphere. Human activities such as agricultural operation, fossil fuel burning, urbanization and manufacturing are responsible for the increase of these gases. As part of the global economy, GHG emissions are a major concern [7,8]. As a result of this, there are no instinctive ways to address the problem. In any individual, group, community, or country of the world it has disastrous impacts on all levels of society. These economies are more sensitive to climate disruption since they have less income. Climate change will therefore have a greater impact on these regions [9,10]. Pakistan is one of the countries’ most vulnerable to climate change, and it has had a negative impact on the economy [11].

With the increase of global economic growth and development over the previous few decades, GHG emissions have increased risen considerably. As a result, low-income countries emit more greenhouse gases than high-income countries. Furthermore, globalization is an important aspect of the economy for the development of a country. Economic progress is unavoidable in the absence of globalization. Nonetheless, there are numerous debates on the subject of globalization. On the one hand, it is widely believed that globalization boosts economic growth and plays an essential role in the country’s development [12]. Globalization, on the other hand, degrades a country’s environment. More notably, globalization has exacerbated the calamity for those economies whose environmental policies are precarious [13].

Globalization has three major dimensions: economic, social, and political. Economic globalization entails the transfer of high skilled labor and technological innovation, capital flows, the eradication of trade restrictions and tariff barriers, colonialism, the flow of goods and services, and the transfer of knowledge across borders. Social globalization entails the interchange of knowledge and innovative fresh ideas among various nations around the world. Furthermore, political globalization aids in the expansion of partnership among countries through a political vision approach [12]. These various aspects of globalization collectively improve socioeconomic indicators such as labor and human rights, poverty reduction, and play an active role in reducing inter-national disputes. Furthermore, cultural differences can be reduced through the flow of exchange of ideas among nations and through people-to-people interactions. Likewise, striving for accelerated economic expansion in all countries, including Pakistan, hastens industrialization, urbanization, transportation, trade, and communication operations. All of these activities and operations place additional strain on energy consumption and power supply [8,14,15]. The energy demand and power supply for industrialization are met by numerous sources such as coal, oil, fossil fuels, and so on. As a result, these phenomena contributes to GHG emissions and worsens the planet’s surface climate and environment. However, these activities existed prior to globalization, but the trajectory in the 20th century, particularly in the last couple of decades, have resulted in the acceleration of global climate deterioration.

Previous researchers have investigated the nexus between globalization and environmental degradation but none of them have approached GHG emissions. For environmental degradation, many researchers have used carbon dioxide as a proxy variable. Factually, carbon dioxide emission is one of the partial factors responsible for environmental degradation. In the context of this research study, the most similar study has been conducted by Khan and Ullah [8] for Pakistan, Shahbaz et al. [16,17,18,19] for Turkey, India, China, and Japan respectively to establish the relationship between globalization and carbon dioxide emissions. Solarin et al. [20] investigated the impact of globalization on air pollution in the case of Malaysia. Their findings confirmed that globalization degrades environmental quality in the country. Salahuddin et al. [21] used panel regression to examine the impact of globalization on carbon emissions. For countries in Sub-Saharan Africa, the study’s findings demonstrate that globalization has a detrimental impact on environmental degradation. Recently, Zafar, et al. [22] investigated the effect of globalization and financial development on carbon emissions in the selected countries of organization for economic cooperation and development. Their outcomes of the study confirmed that globalization and financial development enhance environmental quality. Khan et al. [23] investigated the relationship between globalization, economic growth, energy consumption and carbon emission in South Asian countries. The result of the study reveals that globalization, economic growth, energy consumption has a positive impact on environmental degradation. Nurgazina et al. [24] investigated the impact of globalization, economic growth, and other macroeconomic variables on carbon emission in the case of Malaysia. According to the study’s findings, the results exhibit a positive relationship between globalized economic growth and carbon emissions. Globalization therefore plays a crucial part in the destruction of the environment. Shahzad et al. [25] confirmed that financial development, trade openness and energy consumption cause carbon emission in Pakistan. Further, in the case of Pakistan, Mirza and Kanwal [26] confirmed that energy consumption and economic growth are responsible for environmental degradation in the shape of causing carbon dioxide emission in the country. Dar and Asif [27] revealed that energy use and financial development cause carbon dioxide emissions in India. Similarly, Chandia et al. [28] investigated the relationship among carbon dioxide emission trade openness, economic growth, energy consumption and FDI in the case of Pakistan. The author of the study concluded that these variables are responsible for the deteriorating environment.

Against the drop back of the literature, it is confirmed that globalization has a close association with carbon dioxide emissions. Carbon dioxide is a major element of GHG emissions. However, the rest of the elements such methane, nitric oxide, ozone and F gases [6] are a considerable amount of GHG emissions. Encompassing all of these gases including carbon emission none of the researchers have used GHG emission as a dependent variable in the study. Therefore, we have taken GHG emissions as a dependent variable that has an immense role in the degradation of the environment. Besides, to the utmost knowledge and ability, we confirmed that this is a novel approach to study the impact of globalization on GHG emissions. In addition, all of the researchers have to use an asymmetric approach to investigate the relationship between globalization and environmental degradation and we have used an asymmetric approach along with a symmetric approach.

As a result of this research, Pakistani GHG emissions have been linked to both symmetric and asymmetric as gateways with globalization. The modern time series linear and non-linear ARDL model has been used to achieve the objective of the study. All the preliminary and pre-requisite tests were employed to avoid the unauthenticity of the methodology. The remainder of the paper is premeditated as follows. The next section is methodology. The results and discussions have been presented in the third and fourth sections of the paper. The last section concludes the results.

### Methodology

This study examines the relationship between globalization and Pakistan’s GHG emissions from 1992 to 2017 in Pakistan. Owing to the multidimensional nature of globalization, the KOF Index sub-dimensions are used as independent variables in this study. Globalization on the economic, social, and political levels are among them. The data for these sub-dimensions of globalization were collected from the KOF index. The indices of KOF globalization are within the range from 0–100. The greater value of KOF globalization means a country has higher globalization [29]. The study includes the GDP per capita as an independent variable to avoid the misspecification of the model analysis. This variable data was taken in constant 2010 US $ from World Development Indicators [30]. The dependent variable GHG emissions were measured in metric tonnes. The GHG emissions data was taken from the climate data platform of the climate watch website [31].

To established and assess the relationship between globalization and GHG emissions in Pakistan, the following generalized Equation (1) log-transformed model was employed. The log model has several advantages, for instance, provides reliable estimates, shrinks, normalize, and minimizes the sharpness of the data. Additionally, the coefficients of the variables in the model can be treated as direct elasticities [11,32].

(1) $${\mathrm{LnGHG}}_{t}=\beta_{0}+\beta_{1}{\mathrm{LnGDP}}_{t}+\beta_{2}{\mathrm{LnEGI}}_{t}+\beta_{3}{\mathrm{LnSGI}}_{t}+\beta_{4}{\mathrm{LnPGI}}_{t}+\varepsilon_{t}$$

In Equation (1) LnGHG emissions indicate greenhouse gases, LnGDP is the gross domestic product per capita, LnEGI, LnSGI, and LnPGI signify economic, social, and political globalization Index, respectively. Further, to analyses the asymmetric impressions the Equation (1) independent variable is decomposed into positive and negative changes. The asymmetric presentation of the model is given as follow:

(2) $$\begin{array}{c} \mathrm{Ln}\Delta {\mathrm{GHG}}_{t}=\beta_{0}+\beta_{1}\mathrm{Ln}\Delta {\mathrm{GDP}}_{t}^{+}+\beta_{2}\mathrm{Ln}\Delta {\mathrm{GDP}}_{t}^{-}+\beta_{3}\mathrm{Ln}\Delta {\mathrm{EGI}}_{t}^{+}\\ +\beta_{4}\mathrm{Ln}\Delta {\mathrm{EGI}}_{t}^{-}+\beta_{5}\mathrm{Ln}\Delta {\mathrm{SGI}}_{t}^{+}+\beta_{6}\mathrm{Ln}\Delta {\mathrm{SGI}}_{t}^{-}\\ +\beta_{7}\mathrm{Ln}\Delta {\mathrm{PGI}}_{t}^{+}+\beta_{8}\mathrm{Ln}\Delta {\mathrm{PGI}}_{t}^{-}+\varepsilon_{t} \end{array}$$

**Figure 1:**
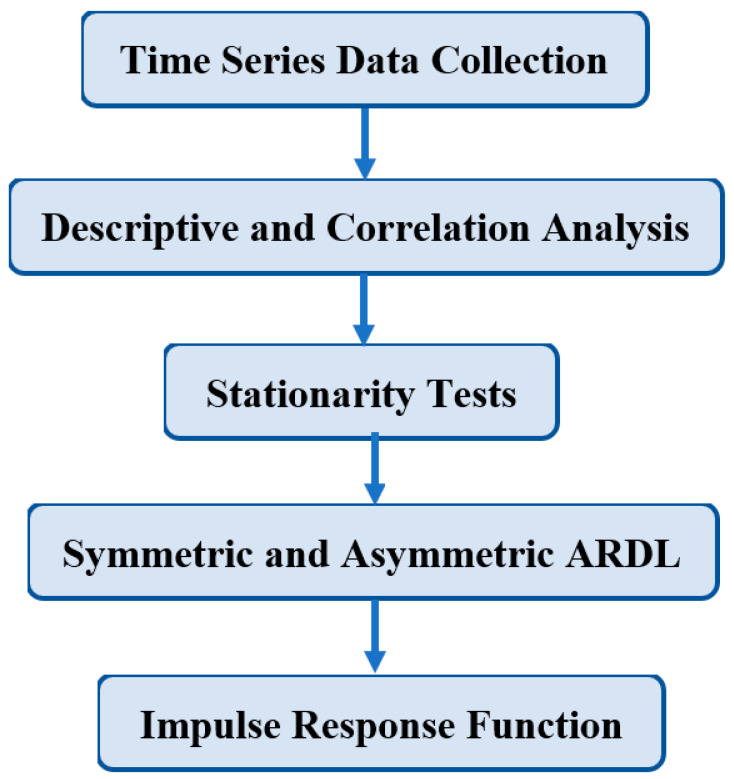
The flow path of methodology.

In the above model, the alternative positive and negative signs of the independent variables represent asymmetries in the model. Figure 1 presents the flow path of methodology.

The annual data has been collected from 1990 to 2017. The pre-condition for the time series analysis is the unit root test. After the confirmation of the integration order of the unit root test, we have chosen the ARDL model and causality to evaluate the symmetric (linear) and asymmetric (non-linear) nexus between globalization and GHG emissions. Therefore, we employed the symmetric and asymmetric ARDL technique which is compatible with sample size. This technique has very relaxed and flexible criteria and may be carried out if the series is I (0) or I (1) or integrated in mixed order. For the execution of the ARDL model appropriate lag length selection is necessary. The appropriateness in lag selection fixed the issue of endogeneity. The lag length pertinence is important particularly in the asymmetric ARDL approach because it smoothly avoids the multicollinearity problem [33]. The ARDL model is a well-known technique because it produces short and long-run elasticities. The ECT term provides likely evidence about the validity of long-run equilibrium. The symmetric ARDL form is expressed below which is transformed from Equation (1).

(3) $$\begin{array}{c} {∆(\mathrm{LnGHG})}_{t}=\delta_{0}+\sum_{q=1}^{r}\beta_{\mathrm{lq}}{∆(\mathrm{LnGHG})}_{t-q}+\sum_{q=1}^{r}\beta_{\mathrm{mq}}{∆(\mathrm{LnGDP})}_{t-q}+\sum_{q=1}^{r}\beta_{\mathrm{nq}}{∆(\mathrm{LnEGI})}_{t-q}+\sum_{q=1}^{r}\beta_{\mathrm{oq}}{∆(\mathrm{LnSGI})}_{t-q}+\sum_{q=1}^{r}\beta_{\mathrm{pq}}{∆(\mathrm{LnPGI})}_{t-q}\\ {+\beta }_{1}{\left(\mathrm{LnGHG}\right)}_{t-1}{+\beta }_{2}{\left(\mathrm{LnGDP}\right)}_{t-1}{+\beta }_{3}{\left(\mathrm{LnEGI}\right)}_{t-1}{+\beta }_{4}{\left(\mathrm{LnSGI}\right)}_{t-1}{+\beta }_{5}{\left(\mathrm{LnPGI}\right)}_{t-1}+\lambda_{0}{\mathrm{ECT}}_{t-1}{+\mu }_{t} \end{array}$$

In Equation (3) the first difference operator is displayed with ∆, the error term is shown with μ_t_. The coefficient of short-run is indicated with β_l,m,n,o,p,q,_ and long-run are presented with β_1,2,__3,4,5_. The AIC lag length selection criteria were chosen to apply the ARDL model. The bound test (F statistics) detects the cointegration among the variables. After the confirmation of cointegration, the short-run and long-run elasticities are determined with the application of the symmetric ARDL model. The independent variables in Equation (2) have provided positive and negative changes. Moreover, the specification of the partial sum of positive and negative changes in the independent variables is expressed below.

(4) $$\begin{array}{l} {\mathrm{LnGDP}}^{+}=\sum_{i=1}^{t}{∆\mathrm{LnGDP}}_{i}^{+}+\sum_{i=1}^{t}{\max(∆\mathrm{LnGDP}}_{i}{,0)\;\mathrm{LnGDP}}^{-}=\sum_{i=1}^{t}{∆\mathrm{LnGDP}}_{i}^{-}+\sum_{i=1}^{t}{\min(∆\mathrm{LnGDP}}_{i},0)\\ {\mathrm{LnEGI}}^{+}=\sum_{i=1}^{t}{∆\mathrm{LnEGI}}_{i}^{+}+\sum_{i=1}^{t}{\max(∆\mathrm{LnEGI}}_{i}{,0)\;\;\mathrm{LnEGI}}^{-}=\sum_{i=1}^{t}{∆\mathrm{LnEGI}}_{i}^{-}+\sum_{i=1}^{t}{\min(∆\mathrm{LnEGI}}_{i},0)\\ {\mathrm{LnSGI}}^{+}=\sum_{i=1}^{t}{∆\mathrm{LnSGI}}_{i}^{+}+\sum_{i=1}^{t}{\max(∆\mathrm{LnSGI}}_{i}{,0)\;\;\mathrm{LnSGI}}^{-}=\sum_{i=1}^{t}{∆\mathrm{LnSGI}}_{i}^{-}+\sum_{i=1}^{t}{\min(∆\mathrm{LnSGI}}_{i},0)\\ {\mathrm{LnPGI}}^{+}=\sum_{i=1}^{t}{∆\mathrm{LnPGI}}_{i}^{+}+\sum_{i=1}^{t}{\max(∆\mathrm{LnPGI}}_{i}{,0)\;\;\mathrm{LnPGI}}^{-}=\sum_{i=1}^{t}{∆\mathrm{LnPGI}}_{i}^{-}+\sum_{i=1}^{t}{\min(∆\mathrm{LnPGI}}_{i},0) \end{array}$$

The structure of the asymmetric ARDL model is designed using the well-known approach of Shin et al. [33]. The mathematical expression of non-linear ARDL model has been presented in Equation (5).

(5) $$\begin{array}{c} {∆(\mathrm{LnGHG})}_{t}=\delta_{0}+\sum_{q=1}^{r}\beta_{\mathrm{lq}}{∆(\mathrm{LnGHG})}_{t-q}+\sum_{q=1}^{r}\beta_{\mathrm{mq}}{∆(\mathrm{LnGDP}}^{+}{)}_{t-q}+\sum_{q=1}^{r}\beta_{\mathrm{nq}}{∆(\mathrm{LnGDP}}^{-}{)}_{t-q}+\sum_{q=1}^{r}\beta_{\mathrm{oq}}{∆(\mathrm{LnEGI}}^{+}{)}_{t-q}+\sum_{q=1}^{r}\beta_{\mathrm{pq}}{∆(\mathrm{LnEGI}}^{-}{)}_{t-q}\\ +\sum_{q=1}^{r}\beta_{\mathrm{qq}}{∆(\mathrm{LnSGI}}^{+}{)}_{t-q}+\sum_{q=1}^{r}\beta_{\mathrm{rq}}{∆(\mathrm{LnSGI}}^{-}{)}_{t-q}+\sum_{q=1}^{r}\beta_{\mathrm{sq}}{∆(\mathrm{LnPGI}}^{+}{)}_{t-q}+\sum_{q=1}^{r}\beta_{\mathrm{tq}}{∆(\mathrm{LnPGI}}^{-}{)}_{t-q}\\ {+\beta }_{1}{\left(\mathrm{LnGHG}\right)}_{t-1}+\beta_{2}{{(\mathrm{LnGDP}}^{+})}_{t-1}{+\beta }_{3}{{(\mathrm{LnGDP}}^{-})}_{t-1}+\beta_{4}{{(\mathrm{LnEGI}}^{+})}_{t-1}+\beta_{5}{{(\mathrm{LnEGI}}^{-})}_{t-1}\\ +\beta_{6}{{(\mathrm{LnSGI}}^{+})}_{t-1}+\beta_{7}{{(\mathrm{LnSGI}}^{-})}_{t-1}+\beta_{8}{{(\mathrm{LnPGI}}^{+})}_{t-1}+\beta_{9}{{(\mathrm{LnPGI}}^{-})}_{t-1}+\lambda_{0}{\mathrm{ECT}}_{t-1}{+\mu }_{t} \end{array}$$

Similar to the symmetric ARDL model approach, the bound test in the asymmetric model is estimated using the same approach of the null hypothesis of no cointegration against the alternative hypothesis of cointegration. After performing the cointegration test the asymmetric impact of the series was analyzed. After the application of both linear and non-linear ARDL models, diagnostic tests were performed to check the suitability, appropriateness, and fitness of these models. Further, the Wald test was performed to check the long-run asymmetric impact of both regressors is significant in the asymmetric ARDL model.

Finally, the symmetric impulse response function was employed using the Cholesky method in the context of the VAR system. This function is an essential tool in empirical causal analysis. Further, it has a vital role in the significance of policy effectiveness analysis. This function examines how GHG emissions react over time to the dimensions of globalization impulses. The econometric model of impulse response function can be expressed with the following Equation (6).

(6) $${\mathrm{LnGHG}}_{t}=\alpha +\sum_{i=1}^{k}\beta_{i}{\mathrm{LnGHG}}_{t-i}+\sum_{j=1}^{k}\gamma_{j}{\mathrm{LnGDP}}_{t-j}+\sum_{l=1}^{k}\Phi_{l}{\mathrm{LnEGI}}_{t-l}+\sum_{m=1}^{k}\delta_{m}{\mathrm{LnSGI}}_{t-m}+\sum_{n=1}^{k}\varphi_{n}{\mathrm{LnPGI}}_{t-n}{+\mu }_{1t}$$

Whereas μ denotes the stochastic error term called impulses or innovation in the system.

## 2. Empirical Results

**Figure 2:**
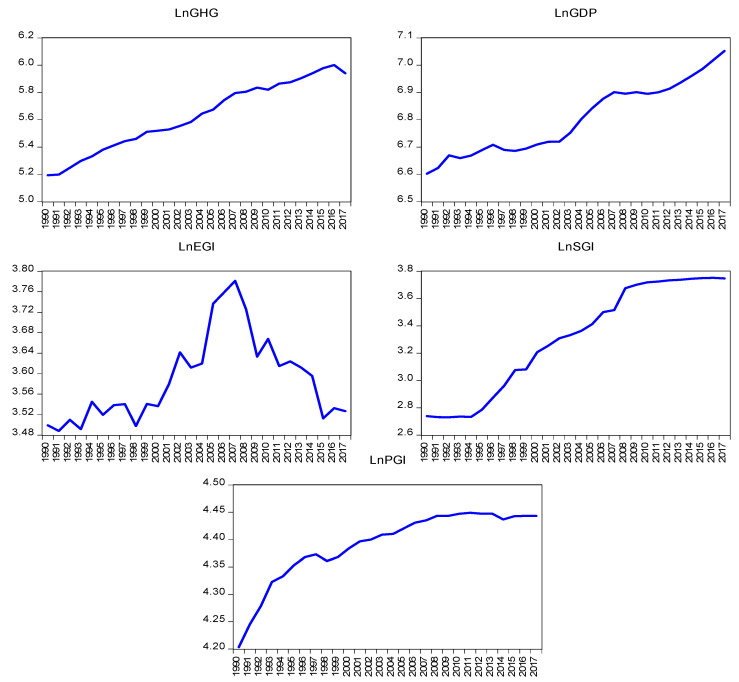
The trend of the series.

Results of the correlation and descriptive statistics are depicted in Table 1. The results of all the explanatory variables show a positive monotonically association with GHG emissions. This means that GHG emissions and all the explanatory variables have a robust association. The descriptive statistics provide the basic features and characteristics of the series and results conclude that all the series are balanced and uniform. Further, the results suggest that all the series data is free from outliers and the nature of the data is normally distributed based on the Jarque-Bera normality test. Figure 2 predicts the all-series trend in the study.

Primarily, the stationarity of all the series was examined with the help of unit root tests. Three different types of unit root tests were performed i.e., ADF [34], PP [35] and Zivote and Andrews [36] as shown in Table 2. All the series are integrated at the first difference I (1). It is important to note that ADF and PP are considered classical unit root tests and often lead to spurious results. It has low power and is considered equivocal. Therefore, the unit root test having structural breaks is considered reliable.

Next, after the confirmation of unit root tests that all the series in the study are integrated of order I (1). In addition, the prevalence structural break in the data has made our job easy to proceed with our analysis. Therefore, we have conducted two pre-requite tests i.e., BDS test Bound tests. First, we have carried out the BDS independent test proposed by Banerjee et al. [37] for the recognition of time series non-linear regression. These test results display that all the series are not identically and independently distributed (Table 3). This means that it is now obligatory to investigate the asymmetric relationship between globalization and GHG emissions. Secondly, once it was ensured that this study has to perform asymmetric ARDL in addition to symmetric ARDL we have checked the bound test for both the symmetric and asymmetric ARDL. The results of both the models are significant at a 1% level of significance as F-statistics in both cases are above the lower and upper critical values. Therefore, these tests indicate that all the variables are co-integrated and obsessed with long-run relationships (Table 4).

The results of the symmetric and asymmetric ARDL models are presented in Table 5&6 respectively. The coefficient of GDP is positive and has a significant impact on GHG emissions both in short and long-run symmetric effects. This means that other things held constant, a 1% increase in the GDP share in Pakistan would release 0.62% GHG emissions. Similarly, in the long-run 1% increase in the GDP share in Pakistan would emit 0.81% GHG emissions. These results corroborate the findings of Khan and Ullah [8]. Further, the results of the dimension of globalization show a positive impact on GHG emissions in the case of Pakistan in both the short and long-run except economic globalization. The negative association between economic globalization and GHG emissions revealed that some mitigating strategies have been adopted while taking foreign investment from abroad. Further, globalization plays a role to improve the climate and environmental condition of the country through income, scale and technique effects. These findings are constant with the results of Shahbaz et al. [16,17]. This means that social and political globalization has a robust association with GHG emissions in Pakistan.

**Figure 3:**
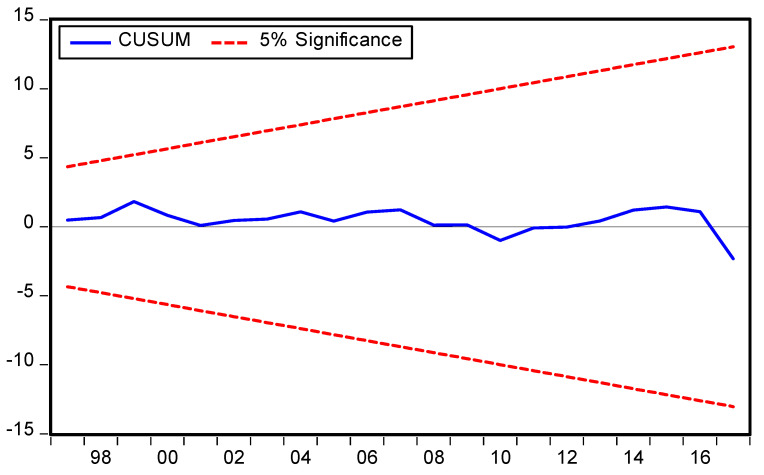
Symmetric ARDL CUSUM test.

**Figure 4:**
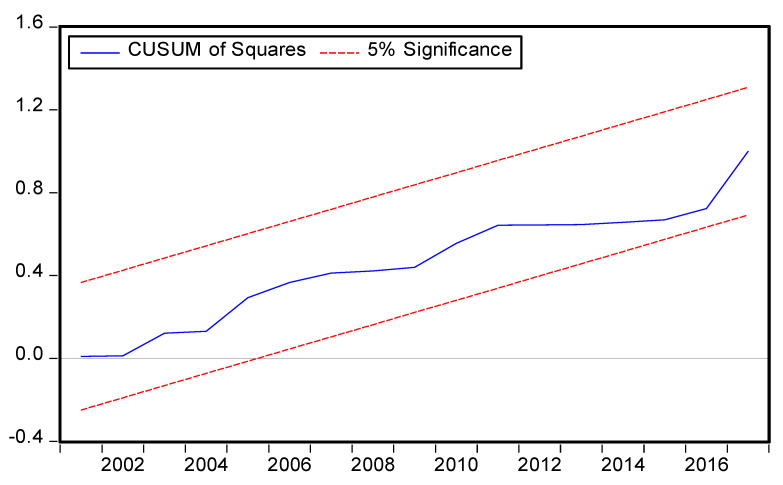
Symmetric ARDL CUSUM Squares test.

**Figure 5:**
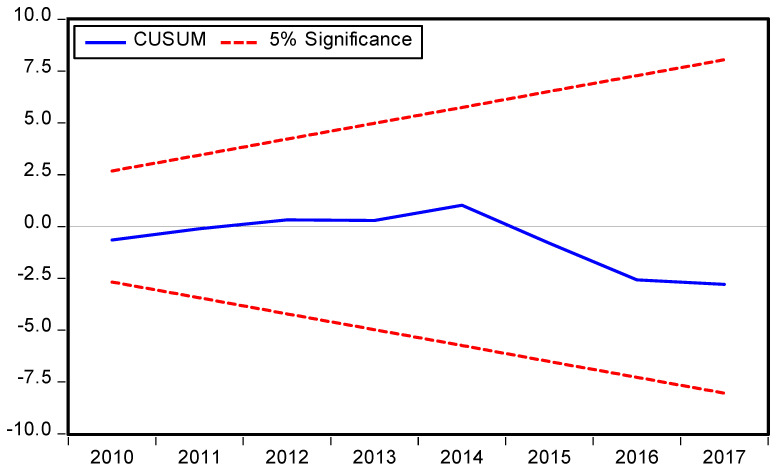
Asymmetric ARDL CUSUM test.

**Figure 6:**
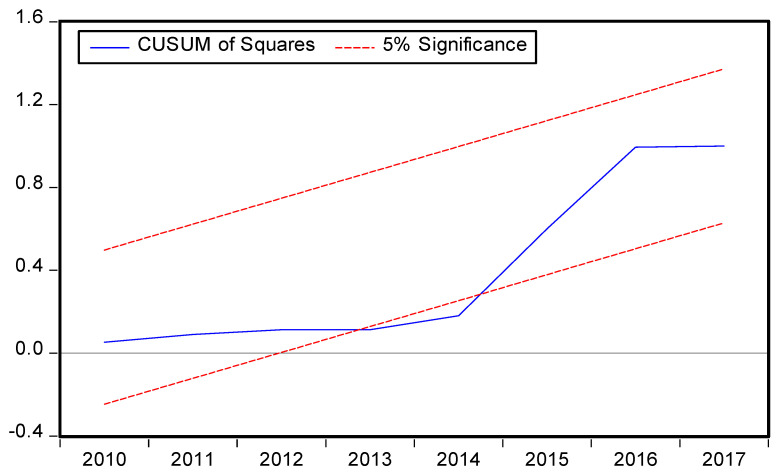
Asymmetric ARDL CUSUM of Squares test.

The symmetric and asymmetric best fitted models criterion is provided in Table 5 and Table 6 respectively and the best fitted models specifications are symmetric ARDL (1,0,0,0,0) and asymmetric ARDL (1,1,1,1,0,1,1,0,1) based on the AIC models selection criteria. The diagnostic tests were performed, and the results vigorously support the current forms of symmetric and asymmetric ARDL models (Table 5 and Table 6). Moreover, the result of χ^2^ RESET test shows that the functional form of the ARDL model is correct having no misspecification, stability (CUSUM), and JB test value with corresponding *p* value shows that the residuals are normally distributed of the symmetric and asymmetric ARDL models are consistent with model specification. This means that the model is free from autocorrelation (χ^2^ LM) and heteroscedasticity (χ^2^ ARCH) problems. The symmetric and asymmetric CUSUM and CUSUM Squares tests are portraits in Figure 3, Figure 4, Figure 5 and Figure 6 respectively.

The results of the asymmetric ARDL model suggest that positive shock of GDP enhances GHG emissions while negative shocks of GDP have a negative impact on GHG emissions, both in the short and long-run in asymmetric effect. These results suggest that a 1% increase in the GDP^+^ enhances 0.54 and 1.09% GHG emissions in the country both in the short and long-run respectively. Further, the results of economic globalization have a consistent result with the symmetric ARDL model in this study. However, the results of EGI^+^ have a positive impact on GHG emissions in the long-run. This means that a 1% increase in the EGI^+^ would bring a 0.33% increase in the impact of GHG emissions. The finding revealed that currently economic globalization does not cause any threat of GHG emissions, but in the long-run, it would create a problem in Pakistan. In short-run, the positive shock of social globalization reduces but, the negative shock of social globalization raises the impact of GHG emissions in Pakistan. These findings are consistent with long-run positive and negative shocks of social globalization results in the asymmetric ARDL model. In short-run asymmetries, the positive shock of political globalization has a negative and significant impact on GHG emissions, but the negative shock of political globalization increases GHG emissions. Nevertheless, in the long-run, the asymmetric effect of political globalization is opposite to the asymmetric effect of political globalization in the short-run.

The error correction term (ECT) in the short-run symmetric and asymmetric ARDL models are negative and significant. This means that the symmetric and asymmetric form of the ARDL model has a long-run equilibrium relationship.

**Figure 7:**
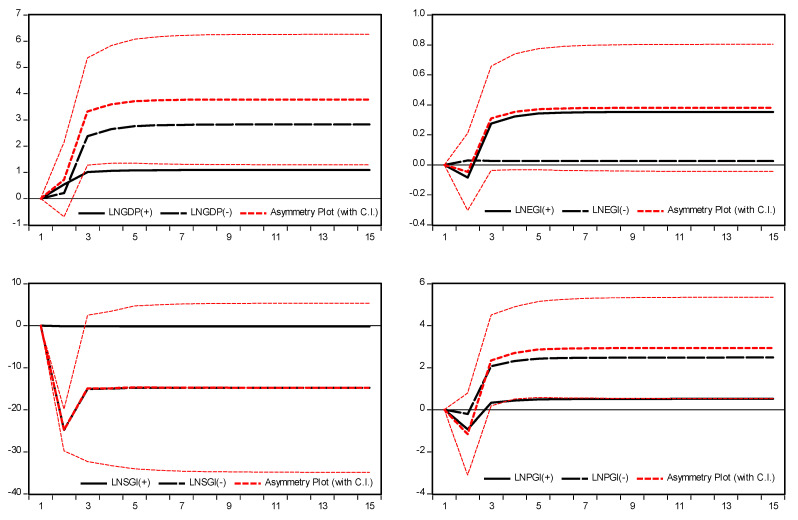
Accumulative dynamic multiplier plots.

Furthermore, to examine the adjustment of asymmetry a dynamic multiplier was analyzed in the prevailing long-run equilibrium. Due to the negative and positive shocks, it would converge to a new long-run equilibrium. Figure 7 presents the accumulative dynamic multiplier plots for GDP and dimensions of globalization. The continuous black line shows how GHG emissions adjust over the horizon due to a positive shock in GDP and dimensions of globalization. The dashed line presents the adjustment of GHG emissions over the horizon due to a negative shock in GDP and dimensions of globalization. The small dash line in the middle is the asymmetric plot which reflects the difference between the dynamic multiplier of positive and negative changes in the GDP and dimensions of globalization. The response of GHG emissions to either positive or negative shock is more prominent in the later stage of the long-run than the short-run. Therefore, GHG emissions respond positively to a positive shock and negatively to a negative shock.

**Figure 8:**
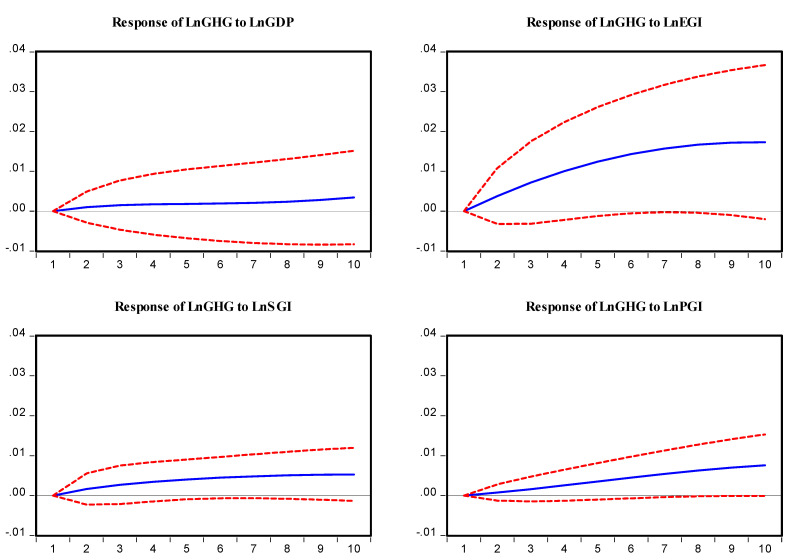
Response to Cholesky one S.D. Innovations ± 2 S.E.

Lastly, this study performs an impulse response function to explain the reaction of an endogenous variable to one of the innovations Figure 8. This function describes the evolutions of the variable of interest along a specified time horizon after a shock in a given moment. Further, it tracks the impact of a variable on other variables in the system. The result of the impulse response function suggests that GDP has a positive relationship with GHG emissions. Initially, it shows a decreasing trend up to 5 periods of horizon after that it slightly shows an increasing trend. This means that GDP would cause GHG emissions in the future more significantly. Economic globalization has a more robust association with GHG emissions. Economic globalization has an increasing trend up to 7 horizons and after that shows a slightly decreasing trend. The impulse response function of social globalization presents flatter innovations than economic globalization. This suggests that it has a positive trend throughout the ten periods of the horizon. Moreover, the outcome of political globalization has a positive relationship with GHG emissions. The innovation in political globalization presents an increasing trend throughout the 10 periods horizon. These results suggest that economic, social, and political globalization would cause GHG emissions in the future in Pakistan.

## 3. Discussions

According to the study’s findings, GDP has a favorable impact on GHG emissions in Pakistan. This result demonstrates that Pakistan’s economic expansion in the early stages of production degrades environmental quality. It is the common consensus among scientists that global long-term climate Changes is caused due to GHGs in the atmosphere. These GHGs are primarily the result of human-caused anthropogenic activities. Furthermore, a country’s rank and status are weighted depending on total production output. This suggests that a country’s GDP fuels human demand. More human needs result in more human activities in the country, which produce more GHGs and inevitably become a source of GHG emissions. In the symmetric ARDL model, the result of economic globalization has a beneficial impact on GHG emissions. Primarily, economic globalization has been involved in global flows of goods and services, foreign direct investment (FDI), and technology transfers in many sectors of the economy such as agriculture, industry, telecommunication, automobile, renewable and non-renewable energy. These activities are the major sources of GHGs emissions in the atmosphere causing environmental degradation and pollution. However, these findings have contradictory results with the asymmetric ARDL model in this study. The co-efficient of positive and negative shocks of economic globalization in asymmetric ARDL suggest that the increasing economic activities as a result of economic globalization would reduce the impact of GHG emissions. ARDL’s symmetrical model does not devolve into an asymmetrical effect, making these conclusions results reliable and robust. Comparing the findings of the two methods, the asymmetrical method yields more unbiased results. This can be clarified with the help of a well-known example. When two or more countries are involved in economic globalization, that time effective policies, practices and global regulatory systems are identified. These rules and regulations are set for controlling economic globalization and for a better sustainable environment by not harming their partner country’s territory. The results of social and political globalization have a positive impact on GHG emissions both in the short and long-run of the symmetric ARDL model. Haseeb et al. [38] argued that globalization enhances environmental degradation and GHGs emissions. However, these results are incompatible with asymmetric ARDL model assumptions. This means that social globalization helps in educating people about the importance of the environment by making them more aware of its importance to them. The positive impact of political globalization in the symmetric model suggests that political globalization creates problems in the economy. In such circumstances internationalization would be avoided and nationalization would be strengthened to protect the environment from degradation. Environmental degradation and pollutions problems can be extinct by introducing more global environmental regulations and policies in the country [39]. Further, political globalization improves the environment because the political government in many industrial countries focuses on national products. The state-level policies and nationalization would reduce contributions to GHG emissions and protect the environment from degradation on global levels [40]. Political globalisation is intrinsically tied to modernism, which is to blame for GHG emissions [41]. Political globalization must be prioritized in the country, and the issue of GHG emissions must be addressed. As a result, this issue can be addressed more effectively by strengthening the role of technocratic government within the existing political system.

## 4. Conclusions

The purpose of this research was to look into the symmetric and asymmetric link between globalization and GHG emissions in Pakistan. The ARDL model was used for this purpose, and time series data was obtained from 1990 to 2017. The time series requirement tests demonstrate that all of the series were stationary at first difference. Also, the stationarity was conducted using various unit root tests such as ADF, PP, Zivot, and Andrews. The symmetric and asymmetric ARDL models produce distinct outcomes. The symmetric results of the ARDL model present that economic, social, and political globalization along with the GDP have a positive relationship with GHG emissions both in the short and long-run. Furthermore, the results of the asymmetric ARDL model reveal that both positive and negative GDP shocks have a positive and negative influence on GHG emissions in the short and long run. Nonetheless, the positive and negative shocks of economic globalization have a significantly negative impact on GHG emissions in the short run. In the long run, the asymmetric ARDL model indicates that the positive and negative shocks of economic globalisation have a positive and negative relationship with GHG emissions, respectively. Furthermore, in the short-run asymmetric ARDL model, the positive shock of social and political globalisation has a negative link with GHG emissions, whereas the negative shock has a positive impact on GHG emissions in the country. Whereas in the long run, the positive and negative shocks of social globalization have a negative and positive influence, respectively, whereas political globalization has a positive and negative relation with GHG emissions in Pakistan. The impulse response function demonstrates that GDP and the scale of globalization have a causal link with GHG emissions in Pakistan in both the short and long run.

The government of Pakistan has already taken initiatives to reduce the impact of high GHGs. Therefore, Eco-system Restoration Initiative (ESRI) were taken to support the environment through ecologically targeted initiatives. These initiatives encompass of afforestation, biodiversity conservation, enhancing policy environment consistent with the objectives of Pakistan’s Nationally Determined Contribution (NDC) and attaining Land Degradation Neutrality (LDN). In the same way “Eco-system Restoration Fund (ESRF)” has been introduced to finance and support the climate change and environmental related projects and program in Pakistan.

Some major policy implications arose from the study’s findings. The findings of this study demonstrated that globalization, both symmetrically and asymmetrically, has robust linkages with GHG emissions. As a result, it is evident that globalization and GHG emissions are incompatible. The government must adopt sustainable environmentally friendly GHG emissions reduction strategies during the production, manufacturing, and transportation of goods. This can be accomplished by signing a memorandum of understanding for a multinational investment treaty which includes the industrialized advanced countries. Furthermore, launching new GHG emissions projects and regulating environmental policies with the cooperation of industrialized advanced countries. Furthermore, it is necessary for the government to introduce enhanced green technology rather than resorting to traditional technologies, introducing innovation in research and development, capital flows, and global industrial networks. In the country, the use of fossil fuels energy should be replaced with renewable energy. This alternate energy consumption would aid in lowering the country’s environmental costs and tackling the concerns of GHG emissions. With the support of social globalization, awareness of GHG emissions concerns and environmental issues should grow. As a result, global institutions should take an active part in mitigating the problem of GHG emissions through political globalization. Ultimately, the government should develop a clear globalization strategy in which it reduces GHG emissions. This can be successfully accomplished by incentivizing the positive impacts of globalization and prohibiting the negative repercussions of globalization through substantial financial fines.

## Figures and Tables

**Table 1:** Correlation and descriptive analysis of the study.

	GHG	GDP	EGI	PGI	SGI
GHG	1.00	0.98	0.42	0.88	0.98
GDP	0.98	1.00	0.39	0.83	0.95
EGI	0.42	0.39	1.00	0.58	0.47
PGI	0.88	0.83	0.58	1.00	0.88
SGI	0.98	0.95	0.47	0.88	1.00
Mean	285.68	907.79	36.31	80.85	29.37
Median	274.65	878.13	35.25	82.25	28.45
Maximum	403.67	1155.36	43.87	85.55	42.62
Minimum	180.12	736.95	32.72	66.91	15.34
Std. Dev.	70.41	120.61	3.18	5.05	10.70
Skewness	0.11	0.37	0.95	−1.22	−0.02
Kurtosis	1.69	1.89	2.94	3.77	1.44
Jarque-Bera	2.05	2.09	4.18	7.63	2.84
Probability	0.36	0.35	0.12	0.02	0.24
Sum	7999.06	25,418.00	1016.71	2263.87	822.24
Sum Sq. Dev.	133,854.50	392,761.60	272.61	688.44	3093.87
Observations	28.00	28.00	28.00	28.00	28.00

Source: Author self-estimation and calculations.

**Table 2:** Results of unit root test.

Parameters	ADF		PP		Zivot and Andrews	
	Level	1st Difference	Level	1st Difference	Level	Break
LnGHG	−1.65	−3.75 ***	−1.66	−3.79 ***	−2.33 **	2010
LnGDP	0.37	0.31 **	0.38	−3.10 **	−2.34 **	2010
LnEGI	−1.50	−5.19 ***	−1.50	−5.19 ***	−2.28 **	2001
LnSGI	−1.05	−4.37 ***	−0.97	−4.22 ***	−4.16 ***	2010
LnPGI	−7.71	−3.27 **	−7.43	−3.34 **	−5.74 **	2000

Note: *** and ** represent levels of significance at 1% and 5% level, respectively.

**Table 3:** Results of BDS non-linearity test.

Series	D 2	D 3	D 4	D 5	D 6
LNGHG	0.188 ***	0.314 ***	0.395 ***	0.444 ***	0.490 ***
LNGDP	0.162 ***	0.261 ***	0.322 ***	0.355 ***	0.357 ***
LNEGI	0.089 ***	0.154 ***	0.182 ***	0.189 ***	0.179 ***
LNSGI	0.199 ***	0.337 ***	0.432 ***	0.496 ***	0.543 ***
LNPGI	0.191 ***	0.326 ***	0.421 ***	0.496 ***	0.539 ***

Note: The BDS test based on the residuals of a VAR for all chosen variables and D denotes Dimension of the variables. *** represent levels of significance at 1%.

**Table 4:** Bound test for Symmetric and Asymmetric ARDL.

Models	F Statistics Value	Significance Level	Lower Bound Value	Upper Bound Value
Symmetric ARDL	7.97	10%	2.20	3.09
		5%	2.56	3.49
		1%	3.29	4.37
Asymmetric ARDL	6.80	10%	1.85	2.85
		5%	2.11	3.15
		1%	2.62	3.77

Source: Author self-estimation and calculations.

**Table 5:** Results of symmetric ARDL.

Short-Run Elasticities				Long-Run Elasticities			
Variable	Co-Efficient	t-Statistic	*p* Value	Variable	Co-Efficient	t-Statistic	*p* Value
ΔLnGDP	0.62	3.20	0.00 ***	LnGDP	0.81	4.84	0.00 ***
ΔLnEGI	0.15	1.53	0.14	LnEGI	0.11	0.81	0.43
ΔLnSGI	0.59	2.05	0.05 **	LnSGI	1.25	2.94	0.01 **
ΔLnPGI	0.03	0.36	0.72	LnPGI	0.20	2.29	0.03 **
Cointeg Eq (−1)	−0.70	−4.48	0.00 ***	Constant	−5.63	−2.80	0.01 **
Model Selection Criteria				Diagnostics Tests			
Log-likelihood	67.20				JB Normality	6.88 [0.13]	
AIC	−4.53				χ^2^ ARCH	0.36 [0.55]	
BIC	−4.24				χ^2^ RESET	0.98 [0.33]	
HQ	−4.45				χ^2^ LM	0.23 [0.79]	
Adjusted R^2^	0.99						
Model Specification		ARDL (1,0,0,0,0)					

Note: *** and ** represent levels of significance at 1%, 5% and 10%, respectively.

**Table 6:** Results of asymmetric ARDL.

Short-Run Elasticities				Long-Run Elasticities			
Variable	Co-Efficient	t-Statistic	*p* Value	Variable	Co-Efficient	t-Statistic	*p* Value
ΔLnGDP^+^	0.54	5.86	0.00 ***	LnGDP^+^	1.09	5.40	0.00 ***
ΔLnGDP^−^	−0.29	−0.74	0.48	LnGDP^−^	−2.65	−2.06	0.07 **
ΔLnEGI^+^	−0.07	−1.28	0.23	LnEGI^+^	0.33	1.51	0.16
ΔLnEGI^−^	−0.06	−1.01	0.33	LnEGI^−^	−0.03	−0.44	0.67
ΔLnSGI^+^	−0.14	−4.07	0.00 ***	LnSGI^+^	−0.14	−1.62	0.14
ΔLnSGI^−^	24.65	14.57	0.00 ***	LnSGI^−^	13.78	1.29	0.23
ΔLnPGI^+^	−0.90	−4.40	0.00 ***	LnPGI^+^	0.52	1.28	0.23
ΔLnPGI^−^	0.22	0.45	0.66	LnPGI^−^	−2.32	−1.66	0.13
CointEq (−1)	−0.87	−11.96	0.00 ***	Constant	5.34	66.17	0.00 ***
Model Selection Criteria				Diagnostics Test			
Log-likelihood	96.19			JB Normality	0.96 [0.61]		
AIC	−6.17			χ^2^ ARCH	0.10 [0.74]		
BIC	−5.39			χ^2^ RESET	1.18 [0.30]		
HQ	−5.95			χ^2^ LM Test	2.92 [0.11]		
Adjusted R^2^	0.99						
Model Specification		ARDL (1,1,1,1,0,1,1,0,1)					

Note: *** and ** represent levels of significance at 1% and 5% respectively.

## Data Availability

Sources of data used for the study are provided in the text, Upon reasonable request, the corresponding author will provide access to the datasets and materials used for analysis of this article.
